# Montreal Medico-Chirurgical Society

**Published:** 1878-03

**Authors:** 


### MEDICO-CHIRURGICAL SOCIETY.

MONTREAL, CANADA, January 26, 1878.

The President, Dr. F. W. Campbell, in the chair.

Dr. Baynes read his paper on Electrical Therapeutics. The following discussion ensued:

Dr. Reddy related a case of *post partem* hemorrhage, where he used every known means to arrest the flow without avail, until he sent for his "Palmers Apparatus," and used it with perfect success. Also related cases where lumbago had been cured by it; had experienced its good effects in himself. He also spoke of having tried it in tinnitus aurium, constipation, etc.

Dr. Butler said that he had been disappointed in its use in tinnitus.

Dr. H. Howard expressed his interest in the paper, and the great number of cases mentioned in which electricity is said to be beneficial, and expressed the hope that it would do away with the use of medicines to a great extent. Said that in old times he had used it often in tinnitus, but with no good effects.

Dr. Trenholme complimented Dr. Baynes on his paper. Expressed his conviction that electricity would be more largely used in the future. Spoke of its good effects in *post partem* hemorrhage, but thought that hot water injected into the uterus would act with the same results. He was glad that there was such a person as Dr. Baynes in the city, and would be disposed to throw cases in his way.

Dr. Shephard said that hot water had been used in Germany for some time in *post partem* hemorrhage. Expressed his skepticism as to the effects of electricity in it, but has seen it used with success in aphonia.

Dr. Kennedy spoke of paralysis in diptheria. Wished to ask Dr. Baynes about the effect of application of electricity upon the heart.

Dr. Proudfoot said that he had not been much pleased with the results, either in eye or ear cases, from the use of electricity.

Dr. Loverin spoke favorably of the use of electricity.

Dr. F. W. Campbell took exception to the statement, that ergot always did the woman and child harm, whilst electricity never did. He had used ergot very largely for seventeen years, but had never seen any evil effect either to child or mother.

Dr. Reddy had done the same thing.

Dr. Trenholme, also, had never seen any bad effects from electricity.

Dr. H. Howard said that he had once seen a case of rupture of the uterus from the use of ergot in a woman who had previously borne children.

Dr. Baynes, in reply, stated that he had been misunderstood with regard to the action of ergot always doing harm. He said that it occasionally did so.

Dr. H. Howard moved, and Dr. Eddy seconded, a vote of thanks to Dr. Baynes for his admirable paper.

Dr. Nelson related the history of a case of cancer of stomach. *Case.*—A small French Canadian woman, who had a tumor over stomach size of an egg. Eighteen months ago had been in good health. Nine months ago first discovered the tumor herself; had had children; had no history of cancerous disease in any of her family. Drs. Kennedy and Fuller in consultation diagnosed cancer of stomach.

Dr. Loverin proposed a vote of thanks, second by Dr. Trenholme, for the very interesting pathological specimens. *Canada Medical Record.*

